# Elsevier’s approach to the bioCADDIE 2016 Dataset Retrieval Challenge

**DOI:** 10.1093/database/bax056

**Published:** 2017-08-21

**Authors:** Antony Scerri, John Kuriakose, Amit Ajit Deshmane, Mark Stanger, Peter Cotroneo, Rebekah Moore, Raj Naik, Anita de Waard

**Affiliations:** 1 Elsevier Ltd, The Boulevard, Langford Lane, Kidlington, Oxford OX5 1GB, UK; 2 Infosys, Hosur Road, Electronics City, Bengaluru 560 100, India; 3 Search Technologies Corp, 1110 Herndon Parkway, Suite 306, Herndon, VA 20170, USA; 4 Elsevier Inc, Jericho, VT 05465, USA

## Abstract

We developed a two-stream, Apache Solr-based information retrieval system in response to the bioCADDIE 2016 Dataset Retrieval Challenge. One stream was based on the principle of word embeddings, the other was rooted in ontology based indexing. Despite encountering several issues in the data, the evaluation procedure and the technologies used, the system performed quite well. We provide some pointers towards future work: in particular, we suggest that more work in query expansion could benefit future biomedical search engines.

**Database URL:** https://data.mendeley.com/datasets/zd9dxpyybg/1

## Introduction

Our team at Elsevier is working on several information retrieval projects, including Elsevier DataSearch, a search engine for research data that is currently in beta (http://datasearch.elsevier.com). The bioCADDIE 2016 Dataset Retrieval Challenge (https://biocaddie.org/biocaddie--dataset-retrieval-challenge-registration) offered us a rare and exciting opportunity to evaluate and understand search strategies and techniques to help researchers find relevant biomedical research data.

The bioCADDIE Challenge was conducted using a collection of (structured and unstructured) metadata from biomedical datasets, generated from a set of 20 individual repositories. A set of representative example queries for biomedical data that were determined by domain experts, were provided for system development. An evaluation was conducted, using a manually annotated benchmark dataset, that consisted of a held-out set of queries, with relevance judgments for datasets in the provided collection. The datasets were annotated as ‘relevant’, ‘partially relevant’ and ‘not relevant’ to the query (1, 2). The bioCADDIE Challenge presented two unexpected subsequent challenges, namely, the need to parse natural language queries, and judgment sets that were sparsely populated.

For this effort, we developed two parallel approaches: one involved word embeddings; the second centered on tagging both the queries and the data sources with named entities. These entities originated from the National Library of Medicine's controlled vocabulary thesaurus, Medical Subject Headings (MeSH; https://www.nlm.nih.gov/mesh/) and the National Center for Biotechnology Information’s database for gene-specific information, Entrez Gene (https://www.ncbi.nlm.nih.gov/gene). For our submission, we combined the various aspects of the two approaches, compared the results and drew conclusions. In order to improve the assessment of our results, we engaged domain experts to judge the datasets. In this paper, we describe what we did to develop our submission.

## Related work

An important resource in locating biomedical information online is through search engines (3). Vocabulary mismatch is a well-studied problem in the field of information retrieval, i.e. the words in a query do not match the words in the data stored in the underlying search engine. In information retrieval (4), query expansion involves adding new query terms to the initial query in order to enhance its match to the dataset. Candidate terms for expansion are either extracted from external resources, such as ontologies or lexical resources, or from the documents themselves, based on the associations with the initial query words (5–10).

In earlier efforts, Bhogal *et al.* (5) provide a review on the performance of ontology based query expansion that uses external human curated knowledge, whereas Díaz-Galiano *et al.* (6) describe the performance in a specific scientific domain. Pseudo-relevance feedback is one of the earliest methods of query expansion by leveraging the dataset itself (11, 12). Abdul-Jaleel *et al.* (13) describe their pseudo-relevance feedback approach using language modeling and feedback, which achieves the best performance.

Word embedding (i.e. mapping of words in natural language to continuous vectors, which encode the semantic and syntactic regularities between words) has been explored in detail (5, 10, 14–17). These vectors are dense, low-dimensional and real-valued: each dimension represents a latent feature of the word, and the vector is induced using neural networks. Recent work has been successful in using word embeddings to derive similar words to query words, for a given dataset (10, 16, 18, 19).

## Methodology

### Overview

As an overall development environment, we decided to work with Apache Solr and open source software development frameworks, because of familiarity with these systems within our team. We indexed each record, preserving the full set of fields provided, and created a single text field with all values in each document. We produced alternative indexes of the data using basic stop word filters and English stemmer and lemma filters. At the last moment, we noticed that there was some non-English material present in the material: this was not taken into account for our submission.

In our submission to the bioCADDIE Challenge, we developed two parallel streams: one that focused on ontologies or knowledge based techniques, and another that leveraged word embedding, as described in related work. Since we decided to pursue these two directions in parallel, we developed the different components as microservices to allow them to be easily integrated within each stream.

**Figure 1.**
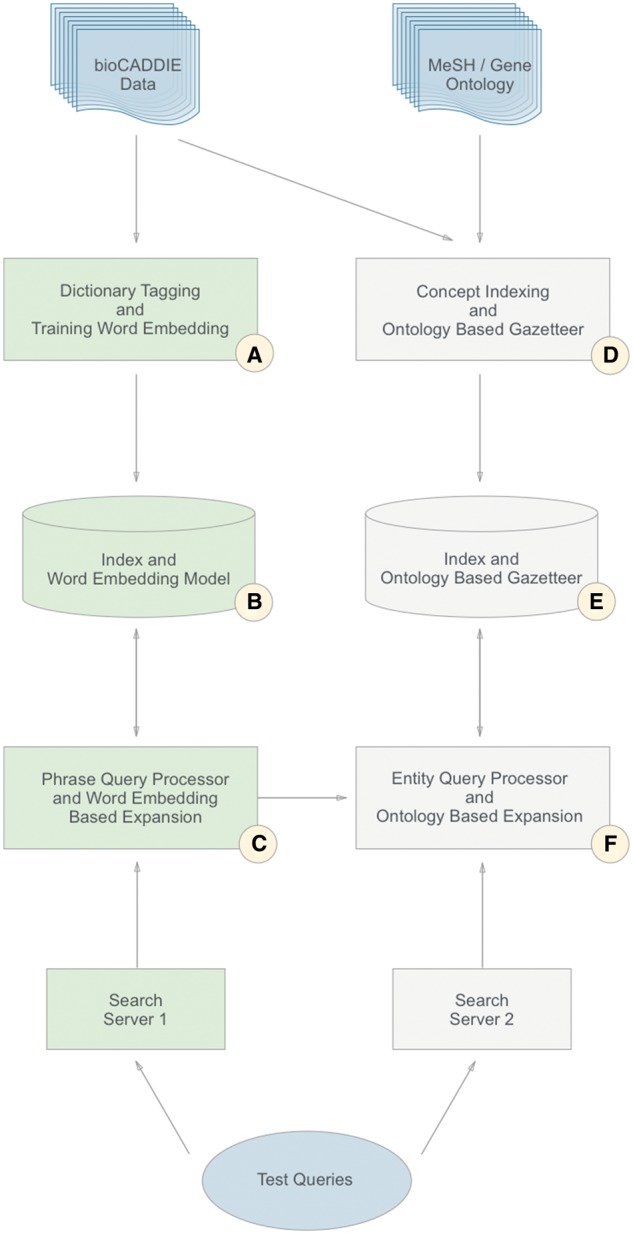
Overview of the two-stream process.

An overview of our architecture, which illustrates the two parallel streams, can be seen in Figure 1. The left-hand stream (A–C) indicates the workflow focused on word embeddings: which is described in Section 1 of the Methodology. The right-hand stream (D–F), indicates the workflow focused on concept indexing, and corresponds to Section 2. In Section 3 of this Methodology, we describe the judging process (B, E). Table 1 describes the match between the sections and the parts of the figure in more detail.

**Table 1.** Mapping from the sections of the Methodology to the elements in Figure 1

Element	Sections
A	1.1, 1.2,
B	3
C	1.3
D	2.1, 2.2
E	3
F	2.3

Our primary focus was not on execution time, and we did not investigate optimizing the implementation. However, from observation of this implementation and other production solutions, the inclusion of query expansion is not likely to add significant time: no more than a delay in the order of tens of milliseconds. Depending on the user query and the degree and type of expansion, the submitted query maybe somewhat larger; however, this falls to the search engine to optimally execute and we would not expect there to be any significant increase in time. The most likely increase would arise from using phrase queries resulting from word embedding expansions. This may also be addressed following the gazetteer approach, by indexing specific concept tokens from a precomputed lexicon, which can then be searched for as a single query term. Attention to the tradeoffs between index time versus query time flexibility can also be adapted to any deployment needs.

The other element that could have an impact is the multi-phase execution plan. Provided the appropriate level of capacity is used to accommodate executing additional searches along with caching, applying them in the order chosen would be able to serve the immediate responses, from the earliest query first. Any overly constrained queries would fail fast, with zero results allowing you to move to the next query without severely impacting the overall response time.

We now describe each of the components of Figure 1, in turn.

## Word embedding and dictionary tagging (a, C)

### Word embedding (a)

Word embeddings are distributed word representations, based on a neural probabilistic language model, where words in a vocabulary are represented as dense real-valued vectors (17). When trained on large text corpora, these models can quantify semantic and syntactic similarities between different words and phrases. For example, the phrases ‘cancer’, and ‘carcinoma’ might be different, yet they are semantically similar, as they occur frequently in a similar context. Word embeddings help capture these similarities.

In this work stream, we evaluated three available libraries for word embeddings [word2Vec, GloVe, fastText, (20–22)] to find a ranked list of similar words and phrases for the purpose of query expansion. The intent was to increase the recall of the search results, by expanding the query with words and phrases that are highly related to the original query words. One characteristic of using word embeddings for query expansion (over a manual dictionary or ontology) is that this allows one to find semantically similar words from the same corpus. To do this, we took the top five phrases related to each phrase given by our word embedding model, and searched for them in the information retrieval index.

Initially, we observed that results obtained by training neural word embedding from word2Vec and GloVe did not improve the normalized discounted cumulative gain (NDCG) metric, compared to the NDCG obtained by using lexicons (20, 21, 23). These models were supposed to give ‘similar’ phrases and words but in fact, they diverged from the main topic of the query. For example, when looking for the top three most similar phrases to ‘glycolysis’, the word2Vec model returned: ‘tca_cycle’, ‘mitochondria_remodelling’ and ‘reroute’, because they occurred in similar contexts in the corpus.

To deal with this problem, we used a recently published technique, ‘fastText’ (22) (https://github.com/facebookresearch/fastText), a library for the efficient learning of word representations and sentence classification based on Facebook research. Instead of training a vector for every distinct word in the corpus, this new approach is based on the skip-gram model, where each word is represented as a bag of character n-grams. A vector representation is associated to each character n-gram, and words are represented as the sum of these representations. This model gave more useful results than word2Vec and GLoVe, and was incorporated into our query expansion.

For example, for the phrase ‘glycolysis’, the top three similar phrases returned by fastText were: ‘gluconeogenesis’, ‘glycolytic’ and ‘glycolytic_pathway’, which are much more satisfactory.

As a further example, if we start with the following example query**:**

‘regulation of DNA repair related to the estrogen signaling pathway in breast cancer patients’

The following tokens were used for expansion:

regulation, dna, repair, estrogen, signaling, pathway, breast, cancer, patients

The Word2Vec Model returns these related phrases:

{‘cancer_drug_resistance’(0.6853), ‘nontransformed’(0.6726), ‘endocrine_cancers’(0.6661), ‘hormone_responsiveness’(0.6610), ‘metastatic_processes’(0.6595), ‘shortterm’(0.6558), ‘lung_cancer_stem_cells’(0.6529), ‘molecular_signature’(0.6518), ‘epidural_anesthesia’(0.6507), ‘adjuvant_treatments’(0.6504)}

The fastText Model returns:

{‘breast_cancer_cell_proliferation’(0.7642), ‘breast_cancer_oncogenesis’(0.7614), ‘tumour_cell_proliferation’(0.7599), ‘iκb’(0.7493613362312317), ‘wilms_tumor_cell_proliferation’(0.7478), ‘breast_cancer_cell_survival’(0.7469), ‘iκbα’(0.745184063911438), ‘breast_cancer_cell_migration’(0.7390), ‘cancer_pathways’(0.7356), ‘breast_cancer_tumorigenesis’(0.7347)}

We observed that character n-grams and smaller window sizes provided better word embeddings for search query expansion. This is similar to the finding by Chiu *et al.* (24) in which intrinsic and extrinsic evaluation of word embeddings were compared. Intrinsic evaluation of word embeddings are compared to human annotations, and extrinsic evaluation uses word embedding as an intermediate step, to evaluate a downstream NLP task like POS, NER, etc. that uses the word vectors. These results reinforce previous findings by Turney (25), conforming that a larger window not only reduces sparsity by introducing more contexts for each word, but is also known to affect the tradeoff between capturing domain similarity versus functional similarity.

Window size 1 gave the best results for extrinsic evaluation while increasing window size to 30 and 50 gave better results for intrinsic evaluation. Window size 1 truly models the word function while increasing the window size trains the word topic function. Essentially, increasing the window size trains association, rather than relatedness. In general, our observations agree with previous work (26) on word space models based on the distributional hypothesis of meaning, though their implementation used a different set of algorithms (LSA and Random Indexing) to derive the context vectors. 

### Dictionary tagging queries (a)

The bioCADDIE dataset provided semi-structured data that was already tagged with important concepts like diseases, genes, organisms, etc. We leveraged the availability of this tagged data to build dictionaries of entity types, based on individual fields in the dataset. This allowed us to detect the set of entity types that matched a phrase in the user query, by running an exact match against this dictionary.

### Phrase query processing (C)

In this stream, we processed the queries to detect chunks of words or phrases, and used a word embedding API to fetch a list of the top five similar words and phrases. These were then used to generate two variants of physical queries that were matched to the index. This process involved segmenting the query in various aspects, as detailed in the following three subsections.

#### Query parsing (C)

The user query was tokenized and passed through a stop word filter. The stop word list was customized with terms taken from the common words found across all queries. The stream of tokens was then passed to a segmenter that looked for prepositions, conjunctions and determiners. The resultant chunks from the user query were then used to find expansions. This involved removing the head and tail of the questions, leaving only the core concepts and interstitial elements to be processed further.

#### Field value tagging (C)

We used the field level information (both values and field names) present in the bioCADDIE dataset to derive custom dictionaries. These dictionaries were then used to tag words and phrases in the query.

#### Query construction (C)

Filtering queries, segmenting and tagging them enabled us to construct three variant forms of the query by applying the following:

- Phrase searching for known sequences of words (derived from field value tagging);
- Expanding words and phrases based on the Word Embedding model trained over the dataset;
- Field based searching based on field value tagging.

The resulting three query forms used were:

*Unigram query (C)*

A unigram query is a simple word-based Solr query without expansion. For this step, we simply took the results of the initial parsing to remove unwanted phrases, and constructed a query with the resulting tokens. The search was based on the aggregated text field.

*Field value tagged query (C)*

Taking the tagged phrases based on values taken from the data, queries were constructed to look for those phrases in specific fields. This improves the previous model by applying phrase searches and field restrictions.

*Field term and embedding expanded query (C)*

In addition to tagging the phrases for querying in specific fields, synonym expansion was incorporated using the fastText word embedding method (22).

As an example, let us consider the following user query:

‘regulation of DNA repair related to the estrogen signaling pathway in breast cancer patients’

Running the query through our Query processor filters out stop words, based on a customized list, and then detects phrase chunks within the query.

The unigram query represents the tokens in the query after stop word removal and becomes:

Query syntax: OR (<unigrams>)

Example query: (value: ((‘regulation’) (‘DNA’) (‘repair’) (‘estrogen’) (‘signaling’) (‘pathway’) (‘breast’) (‘cancer’) (‘patients’)) )

The field value tagged query finds field associations from the schema for each phrase in the query, and becomes:

Query syntax: OR (<Field:Value>) leads to a reformulation of the example query to:

((METADATA.keywords_sField:‘regulation’)^0.5)

OR ((METADATA.citation.journal_sField:‘DNA repair’) ^0.2

OR (METADATA.keywords_sField:‘DNA repair’)^0.5)

OR ((METADATA.StudyGroup.name_sField:‘estrogen’) ^0.2

OR (METADATA.Treatment.agent_sField:‘estrogen’)^0.2)

OR ((METADATA.keywords_sField:‘signaling’)^0.5)

OR ((METADATA.StudyGroup.name_sField:‘pathway’) ^0.2)

OR ((METADATA.StudyGroup.name_sField:‘breast cancer patients’)^0.2

OR (METADATA.Disease.name_sField:‘breast cancer patients’))

## Ontology based tagging and concept indexing (D, F)

For the second indexing stream, we ingested the National Library of Medicine's controlled vocabulary thesaurus, Medical Subject Headings (MeSH), and the National Center for Biotechnology Information’s database for gene-specific information, Entrez Gene.

We briefly investigated other potential solutions for tagging concepts. One challenge was the breadth and depth of the domain and the accuracy of any solution with respect to recall and precision, and, in particular, handling cases of semantic ambiguity. Given the breadth of the domain coverage, and likely depth, in terms of the concepts expressed, this would require extensive reviewing accompanied by the understanding of the full capabilities and nuances of each solution. It would have likely led to an ensemble approach, adding to the complexity of the solution, which was difficult given the time constraints. Also, leveraging online services rather than embeddable processes would have negatively impacted processing times and the turn around times for indexing. We were interested in embedding the concept indexing into the indexing process, which required offset level annotations (as opposed to summary annotations). For simplicity’s sake, we therefore chose to incorporate our own solution, knowing its particular strengths and weaknesses (in particular the domain coverage) to allow us to appropriately use it and assess its impact.

### Ontology based gazetteers (D)

Entity extraction was performed by generating a token stream, and then examining the tokens as they pass through, to see if they matched any of the words and phrases in the MeSH and Entrez Gene dictionaries. Named Entity Tagging was done by taking the respective fields containing primary names, labels, symbols and any alternatives from the ontology sources (MeSH and Entrez) and building gazetteers from them. Commas in the MeSH content were removed, and combinations of terms were added as additional terms. An example of an entry with a comma is ‘Muscle, Abdominal’, which was transformed to ‘Abdominal Muscle’. This increased the chances of correctly matching the phrase in the document text or queries in subsequent applications. Entries with multiple commas were left untreated, as the occurrence of these was rare; all other punctuation, such as parentheses, were left untreated.

Extraction was performed with the following considerations:

- The longest phrase took precedence;
- For MeSH and the case insensitive Entrez Gene terms, the tokens were converted into lowercase before being checked against the dictionaries;
- For the case sensitive Entrez Gene terms, the tokens were kept in their original case;
- No punctuation filters or stemming were applied;
- If a term included a dash in the dictionary, then it required the dash to exist in the JavaScript Object Notation (JSON) document, for it to be recognized.

The longest match precedence rule allowed us to take the most specific form concept mentioned; however, there were still instances where a longer surrounding concept existed, that had not been captured in the dictionaries. Another way to identify classes of entities would be to supplement this dictionary tagging with statistical entity detection, which could then be used to target these concepts as a phrase.

The MeSH dictionary included the majority of the variations, which could be normalized via stemming, so lack of stemming was not an obstacle. However, further improvements could be made by introducing stemming and punctuation filters (hyphenation being a common one) on the JSON text and the dictionary terms.

### Concept indexing (D)

To achieve better retrieval results, we applied a concept indexing scheme, based on named entity tagging. When a MeSH concept or gene was identified within the document text, its concept ID (derived from the source ontology) was then embedded into the text immediately following the relevant term. A custom Solr token filter was used to index the IDs in the same word position as the preceding token in the index. Embedding IDs in this way, rather than just adding them to a specific field, allowed for proximity searches to be applied. It also allowed us to use their frequency with respect to relevancy scoring to enable term frequency–inverse document frequency (tf-idf) techniques.

In addition to adding the IDs of the matched concepts, ancestor concepts (parent, grandparent, etc.) were also embedded, but to differentiate them from the explicit mentions they were marked as implicit concepts. Only MeSH had such hierarchical information: we assumed that the existence of a MeSH concept implicitly meant that it is also related to a parent concept.

There is more work to be done on the use of ontologies to improve the accuracy of the tagging to handle other variations, and provide better semantic disambiguation. We mention a few: abbreviations; spelling variations and special tokens.

#### Abbreviations

We noted that MeSH does not include abbreviated species names (such as ‘M. musculus’), which will therefore be missed. Some of this can be done by word embedding similarity matching, by first processing phrases to look for similar phrases: if we tag ‘M. musculus’ to ‘Mus musculus’ this would yield a match, which would have been a logical next step. This is an example of our trying to strike a balance between curating knowledge and applying machine learning models, to achieve the best result with the resources we had available.

#### Spelling variations and special tokens

Another issue was that ontologies do not always capture spelling variations, such as American English versus British English. Similar issues occur with the use of Greek characters and names (β versus beta) as well as concepts such as ‘stage II breast cancer’, causing problems due to the variation between Roman numerals and decimal numbers (III versus 3). Some of these issues were accommodated by appropriate normalization with character/token mapping in the dictionaries, or the sources being processed.

#### Absence of concepts

Other concepts were not captured in ontologies at all. For example, Entrez Gene does not identify gene collections such as the common BRCA genes, this therefore does not allow for this to be detected and expanded to group BRCA1 and BRCA2 mentions.

A further concern was the difficulty to match passing references to concepts that were not directly related to the data item. This issue can arise simply because one is making a comparison with something else, or more often when one is citing related work. Both situations should not constitute a relevant match. Rather than specifically aiming to identify these through deeper analysis of the text, we hoped to avoid the issue by the looking at the explicit mentions in the specific fields aligned with those concepts and their general frequencies in the records. This method was based on the assumption that there would be more instances that were directly applicable than a passing reference.

Further care in selection ontologies could have yielded more accurate and comprehensive matches; we cannot state for sure that this would have provided any clear benefits.

### Query processing (F)

Taking the natural language queries and constructing a query for the search engine involved removing unwanted segments, identifying and preserving key concepts from the rest. This largely involved segmenting the query based on various annotators:

- Field word matching;
- Exact field value matching;
- Ontology based tagging;
- Dependency parser chunking.

We will discuss the various steps we used in query processing, in turn.

#### Query parsing (F)

As a first step, the user query was tokenized and passed through a stop word filter and parsed against a grammar. The stop word list was customized with terms taken from the common words found across all queries. The stream of tokens was then passed to a segmenter that used grammar to derive common patterns in the queries. The resultant chunks from the user query were then passed on, to find synonyms. This took care of removing the head and tail of the questions leaving the core concepts and interstitial elements to be processed.

#### Named entity tagging (F)

Next, the Named Entity Gazetteers described in 2.1 were used to identify MeSH concepts and genes within the query. The output of the query pre-parsing phase is a list of tagged query elements, which is ordered as follows:

- MeSH concepts annotated with the MeSH concept ID and the IDs of the descendant MeSH concepts;
- Genes annotated with the gene ID;
- Words and phrases identified via field value tagging and annotated with the relevant fields;
- All remaining query terms.

#### Query construction (F)

Next, parsing user queries by segmenting, filtering and tagging them enabled us to construct various forms of the Solr query by taking the following steps:

- Phrase searching for known sequences of words (derived from field value tagging);
- Concept ID searching from named entity tagging and indexing;
- Field/term boosting.

We also conducted some experiments where we applied standard Solr Extended DisMax (eDisMax) query parser, with varying results.

*Concept Expanded Query (F)*

The final method of query processing used named entity tagging. This meant that not only did we have the original and additional similar phrases from word embeddings, we also had the recognized concepts, including inferred concepts based on ontological relations. The component provided a great deal of flexibility in how the various tagged elements could be combined, as well as control over the behavior of the constructed query. This offered many variations which, given a comprehensive judgment set, could have been further explored to find optimal configuration. We targeted a few different combinations which appeared to make the most improvements.

*Expansion Rules*

At a high level, the query consists of a Boolean AND over two main parts: the MeSH and gene concepts in one part, and everything else in the second part. The MeSH and gene part of the query is a Boolean OR across each MeSH or gene identified within the query:

- The MeSH concepts are searched for as a Boolean OR on the following:
  - Original query word(s) as a Boolean AND;
  - Original query words as a phrase (if there are multiple words);
  - The MeSH ID;
  - The MeSH ID (implicit instances);
  - The MeSH ID of each of the descendants in the hierarchy;
  - The MeSH ID of each of the descendants in the hierarchy (implicit instances).
- The genes are searched for as a Boolean OR on the following:
  - Original query word(s) as a Boolean AND;
  - Original query words as a phrase (if there are multiple words);
  - The gene ID.

The remaining part of the query is a Boolean OR across each word or phrase identified through Field Value Tagging plus all other words within the query (other than stop words and natural language elements). If the term is a phrase then it is searched for as a Boolean OR across:

- Original query word(s) as a Boolean AND;
- Original query words as a phrase (if there are multiple words).

The precision of the query was controlled via an API by applying ‘minimum should match’ restrictions to each of the two parts. Additional features which could be controlled via the API include:

- Whether to check for MeSH concepts;
- Whether to include implicit MeSH concept IDs in the query;
- Whether to include descendant MeSH concept IDs in the query;
- Whether to check for gene IDs;
- Boosts to apply to different parts of the query. Different boosts can be applied to each different type of query clause (MeSH ID, MeSH phrase, gene ID, gene phrase, other phrase, Boolean AND of individual phrase words for MeSH, gene or other).

An example would look as follows.

Given the following example user query:

‘Regulation of DNA repair related to the estrogen signaling pathway in breast cancer patients’

The following SOLR query components were created:

1. We used the query parser to identify MeSH concepts and Genes and any other key terms. This query contains no Genes but does contain 5 MeSH concepts.
2. We created a sub-query for each of the MeSH concepts, plus for the other key terms:
  - MeSH Concepts
    - regulation
      - Regulation_Query = (regulation)^20 OR (Explicit MeSH Concepts) OR (Implicit MeSH Concepts)^0.5
    - DNA repair
      - DNA_Repair_Query = (dna AND repair)^0.5 OR (‘dna repair’)^20 OR (Explicit MeSH Concepts) OR (Implicit MeSH Concepts)^0.5
    - estrogen
      - Estrogen_Query = (estrogen)^20 OR (Explicit MeSH Concepts) OR (Implicit MeSH Concepts)^0.5
    - signaling pathway
      - Signaling_Pathway_Query = (signaling AND pathway)^0.5 OR (‘signaling pathway’)^20 OR (Explicit MeSH Concepts) OR (Implicit MeSH Concepts)^0.5
    - breast cancer
      - Breast_Cancer_Query = (breast AND cancer)^0.5 OR (‘breast cancer’)^20 OR (Explicit MeSH Concepts) OR (Implicit MeSH Concepts)^0.5
  - Other key terms
    - patients
      - Patients_Query = patients^2
  - The following words are ignored:
    - of
    - related
    - to
    - the
    - in
3. We created the final query by combining the different query elements as an Boolean OR with a minimum should match parameter to control the precision, leading to the following query:

Regulation_Query OR DNA_Repair_Query OR Estrogen_Query OR Signaling_Pathway_Query OR Breast_Cancer_Query OR Patients_Query

The most precise query requires all sub-queries to be satisfied. Subsequent queries, for the lower parts of the results list, would use a progressively lower minimum match parameter.

#### Multiphase query execution (F)

One area of concern was maximizing the precision of the results. To achieve this, we tried to locate records with all query concepts matched first, followed by series of queries with varying degrees of relaxation (generalization of concepts, specificity of fields to use, relaxing of Solr slop factor on phrases, numbers of clauses which must match, etc.). Each response set was then placed before any subsequent queries, removing results from earlier queries, using a custom service that sends multiple queries to Solr to be processed. Each query gets progressively more relaxed, using various configurable parameters. The aim is to make the most precise results appear at the top of the results list. Queries are sent in parallel to achieve performance. By doing this, we can aggregate the top 1000 results from multiple queries in under a second. 

## Indexing the dataset (B, E)

To collect, manage and use human judgment data against results from our search system, we custom-built a web application that has numerous features to capture human annotation of search results. The web app also allowed us to highlight conflicts between users, merge data and automate the computation of the evaluation metrics, including normalized discounted cumulative gain (NDCG) and precision (23), and integrate our key components**.** It generates a set of variant set of queries, allows users to edit the generated Solr query, and easily compare evaluation metrics for the changed query against previous variants, for a user query. A team of domain experts was hired to provide input into the system, by judging results as ‘relevant’, ‘partially relevant’ or ‘not relevant’.

Overall, we selected 15 queries as our evaluation set. This included the six queries that were provided as part of the bioCADDIE Challenge along with the corresponding judgment data. The rest of the queries were taken from the larger set of initial sample queries provided and two additional queries were created to better suit the expertise of internal judges. Results for each of the queries were judged by internal judges. Judgments were made based on the definitions of ‘relevant’, ‘partially relevant’ and ‘not relevant’ as specified in the challenge.

We asked the domain experts to consider synonyms or alternative expressions which constituted the same concept in the query, so they would not limit themselves to what a baseline system would be able to match. For example, they should include more specific forms of a concept, i.e. ‘diabetes type ii’ in a record for a query on ‘diabetes’ but not more generic forms, i.e. ‘diabetes’ in a record for a query on ‘diabetes type ii’.

Based on the data present in the display, duplicate records were judged individually. Some records had fewer fields or truncated values; if insufficient data was present, they could be judged in the same way as a more complete record. Our final judgment set contained 1532 judgments across 15 queries. However, the largest set per query was only 163. We did find several queries for which no relevant documents were identified; this raised the question of whether this was intentional, if we had missed data, or if there was another issue with the query itself.

We were able to run continuous assessments against the judgment data as we progressed, but the breadth and depth of the coverage we were able to achieve did not provide satisfactorily stable results. Given more time and effort, we would be able to provide a more useful corpus, for future evaluations. 

## Results

For our submissions, we ran the end-to-end system, starting from the full natural language queries, to provide automatic runs through two different setups. To improve the results, we then modified the queries in different ways, notably:

1. By expanding abbreviated species names, e.g. ‘M. musculus’ to ‘Mus musculus’, and ‘D. melanogaster’ to ‘Drosophila melanogaster’;
2. By replacing Greek characters, e.g. ‘β’ to ‘beta’ and ‘κ’ to ‘kappa’;
3. By removing additional stop words, and interstitial phrases and trailing ‘s’.

In retrospect, these issues could have been dealt with as follows:

1. If we had augmented the ontological data sources to incorporate the necessary variations we could have dealt with these issues automatically. Although we spotted these issues early on, resolving them would have meant incorporating full ontologies, not just individual cases, as we did not know which instances would occur in the final test queries. We therefore chose to address these by modifying the queries as necessary;
2. Greek characters could also have been handled with mapping files;
3. The removal of additional terms aimed to reduce some noise observed in other elements of our components; this could have been done automatically, as well, but the effort would have been more than treating this on a case by case basis.

We uploaded five runs in our submission. The definitions, components and ordering of runs 1–5 were as follows:

1. Using **full queries** as provided with the natural language query processor followed by unigram query construction against **an un-stemmed/lemmatized index**, using the single aggregated text field (automatic);
2. Using **full queries with slight modification** (abbreviation expansion on species names and replacing of kappa character with letter k) and with field value tagged plus **embedding expansion query**, against the **un-stemmed/lemmatized index** (manual);
3. Using the **full queries**, applying a default configuration of the concept expanded query and multiphase query execution against a **stemmed index** (automatic);
4. Using **modified queries** (extra terms removed, abbreviated species names expanded, kappa character replaced with k) applying the concept expanded query and multiphase query execution against a **stemmed index** (manual);
5. Using **modified queries** (same as 4) and concept expanded queries but **with no descendants** included and setting term boosts to 1, and without multiphase query execution against a stemmed index (manual).

The results for our submission are shown in Table 2. Our submission was scored along with all other participants using metrics that were defined by bioCADDIE. In all ten groups submitted, up to five runs covered all 15 test queries. Our fourth run achieved first place for both P@10 ± partial measures and NDCG@10 measure, and this is likely attributed to the use of the multiphase query.execution. It scored eighth out of all runs (fourth in terms of top runs from each group) for infNDCG and eighth for infAP. Our fifth submission did slightly better on infAP, scoring third in all runs (second in terms of top runs from each group).

**Table 2.** Results for our submissions as calculated by bioCADDIE using the inferred scoring scheme (27)

Run	Submission	infAP	infNDCG	NDCG@10	P@10 (+partial)	P@10 (-partial)
1	elsevier1.txt	0.2789	0.4292	0.5271	0.7	0.2667
2	elsevier2.txt	0.2963	0.3925	0.5242	0.7067	0.2667
3	elsevier3.txt	0.281	0.4219	0.5514	0.7133	0.3667
4	elsevier4.txt	0.3049	0.4368	0.6861	0.8267	0.4267
5	elsevier5.txt	0.3283	0.4235	0.6011	0.7133	0.34

Table 2 only shows the final submission runs and their subsequent evaluation scores as produced by the final judging rounds. We ran several additional configurations, the numbers based on the limited judgment data we had at the time and only gave general trends largely based on the two approaches. We were unable to select the final configurations purely on those numbers, so we had to also rely on a degree of intuition based on observations. Of course, this leaves open the opportunity for additional work to explore the parameter space. Unfortunately, we did not have time to retrospectively score those runs using the final judgment data made available and are therefore unable to include any additional data, at this time.

We did not observe an improvement in our relevance scores by the use of query expansions from word embeddings. Our runs showed that such extrinsic evaluation of embeddings in search requires much tuning to get a high-quality set of related words and phrases. Given the time we had, we did not tune the embeddings to obtain improved relevance scores. However, we also believe that this represents only one way of applying embeddings in search. Other applications include using embeddings to obtain aggregate vectors for documents and queries, and using a distance metric to drive search ranking. 

## Discussion

### Observations and challenges

During the process of developing and running these pipelines, we encountered a few obstacles, which are now described (with topics highlighted in each paragraph).

The sample queries for the bioCADDIE Challenge ranged from the simple identification of a disease (e.g. a disease in quotes) through to a single phrase, encapsulating several specific expressions that describe it. In some cases, however, we were not able to address the second scenario, due to a **lack of domain knowledge** in the development team.

Another challenge we did not have time to address was **negation** in the data. Interestingly, these negations were not expressed in the queries themselves, which would have added a further level of complexity. Negation can appear in explicit instances such as the exclusion criteria for a clinical study, which is often just expressed as free form text rather than structured data.

A subtler problem presented itself when **contradictory information** was presented. For instance, assigning ‘Mus musculus’ as a species hit, would negate ‘Human’ as a possible match. This case is somewhat avoided by the natural ranking pushing those with matching mentions of the required concept above those without. However, identifying such contradictory information and querying against it could provide more explicit filtering.

The natural language used to express concepts caused a problem when phrases included **conjunctions**, because it obscured entities inside a regular expression. An example: ‘stage I, II and II breast cancer’ needs to be interpreted as ‘stage I breast cancer’, ‘stage II breast cancer’, ‘stage III breast cancer’. This was further compounded by terms being either simple adjectives or parts of a proper name.

**Semantic disambiguation** of concepts also posed other challenges, e.g. a gene being called ‘anxiety’ (https://www.ncbi.nlm.nih.gov/gene/493091) requires a deeper understanding of the text than simply matching the sequence of words.

During search matching we did not leverage specific fields for **known entity types**, which could have yielded more specific matches. For example, when identifying an organism, we could have restricted the higher boosting of results to those with ‘species’ field values, as these are explicit mentions of the related concepts. This would have distinguished them from the results with mere mentions in other fields.

Although **proximity boosting** is supported by the toolset we used, we did not use it as a means for approximating relations between concepts (i.e. the closer two concepts are, the more they are related). This tool could be applied in combination with artificial gaps in the token indexing, by spacing out sentences, paragraphs and separate fields (in the case of an aggregate field). In the case of aggregated fields, keeping related hierarchical fields closer together would have been more useful than mixing fields in some random order.

**Pseudo-relevance feedback** is an unsupervised method for extracting keywords for query expansion. We took the top results for an existing query as feedback, and extracted a set of keywords from this document set, that were then used to expand the query. However, in our measurements we did not observe any improvement on the underlying NDCG score and therefore we did not include this method in our submission. We could have experimented with variations of the approach in how the feedback documents and the candidate keywords are selected.

### Future work

In summary, although we were quite satisfied with our resultant rankings, there are a few points which would have improved our submission, and which we would like to address in the future.

Initially, we considered directly **indexing the data items** associated with the metadata records, which we are doing within Elsevier DataSearch. However, apart from costing a significant greater amount of development resources, we were concerned that there would be a time gap between different versions of the data and metadata provided. Also, it was unclear if other teams would be querying the same primary data. Because we have experience in improving results when full datasets are indexed, this is worth following up on in the future.

Secondly, we would have liked to incorporate **deeper information extraction techniques** and **alternative knowledge representations** in order to better match the concepts expressed in the queries against those in the data.

When covering such a wide domain as biomedical science, it takes time and care to prepare domain knowledge expressed as ontologies and other knowledge databases, to provide a comprehensive coverage of the entire domain and address semantic disambiguation issues. **More use of word embeddings** in combination with an edit measure for handling spelling variations and errors would fill these and other gaps. Further work on concept proximity, as a basic form of relatedness, would undoubtedly also improve the system, and the functioning of the model which targets portions of queries, assigns them to specific fields, and expresses the entity class in question would be refined. Standardizing the entire corpus to such a data model to provide consistency is no small effort, however.

Overall, our results do show some degree of improvement over bioCADDIE’s baseline system. At the time, we were unable to effectively explore all hyper-parameter tuning and configurations due to the sparsity of judgment data available. Our efforts to increase the judgment data may have been biased due to the expertise of judges in relation to the queries being addressed, and may have been influenced by the order in which the data was presented for judging, leading to a lack of resources and time.

Now that the judgment data is available from the bioCADDIE Challenge evaluations, we would like to investigate this issue in the future to fully explore some of the parameter space. In addition, a more in-depth analysis of the use of fastText for query expansion in the biomedical domain is needed, as it has not yet been studied in detail, nor reported in literature. This would further enable us to handle the true nature of a ‘concept’ and how it can be expressed in text, which we believe to be an interesting topic of study, and one that can bring improvements in the field of biomedical information retrieval. 

## Funding

The bioCADDIE 2016 Dataset Retrieval Challenge was supported by NIH grant U24AI117966.

*Conflict of interest*. None declared.
